# Antioxidants green tea extract and nordihydroguaiaretic acid confer species and strain-specific lifespan and health effects in *Caenorhabditis* nematodes

**DOI:** 10.1007/s11357-023-00978-0

**Published:** 2023-11-04

**Authors:** Stephen A. Banse, Christine A. Sedore, Erik Johnson, Anna L. Coleman-Hulbert, Brian Onken, David Hall, E. Grace Jackson, Phu Huynh, Anna C. Foulger, Suzhen Guo, Theo Garrett, Jian Xue, Delaney Inman, Mackenzie L. Morshead, W. Todd Plummer, Esteban Chen, Dipa Bhaumik, Michelle K. Chen, Girish Harinath, Manish Chamoli, Rose P. Quinn, Ron Falkowski, Daniel Edgar, Madeline O. Schmidt, Mark Lucanic, Max Guo, Monica Driscoll, Gordon J. Lithgow, Patrick C. Phillips

**Affiliations:** 1 Institute of Ecology and Evolution, University of Oregon, Eugene, OR 97403 USA; 2 Department of Molecular Biology and Biochemistry, Nelson Biological Laboratories, Rutgers University, Piscataway, NJ 08854 USA; 3 The Buck Institute for Research On Aging, Novato, CA 94945 USA; 4 Division of Aging Biology, National Institute On Aging, Bethesda, MD 20892-9205 USA

**Keywords:** *Caenorhabditis elegans*, *Caenorhabditis briggsae*, *Caenorhabditis tropicalis*, CITP, 17α-estradiol, Acarbose, Green tea extract, Nordihydroguaiaretic acid, Rapamycin

## Abstract

The *Caenorhabditis* Intervention Testing Program (CITP) is an NIH-funded research consortium of investigators who conduct analyses at three independent sites to identify chemical interventions that reproducibly promote health and lifespan in a robust manner. The founding principle of the CITP is that compounds with positive effects across a genetically diverse panel of *Caenorhabditis* species and strains are likely engaging conserved biochemical pathways to exert their effects. As such, interventions that are broadly efficacious might be considered prominent compounds for translation for pre-clinical research and human clinical applications. Here, we report results generated using a recently streamlined pipeline approach for the evaluation of the effects of chemical compounds on lifespan and health. We studied five compounds previously shown to extend *C. elegans* lifespan or thought to promote mammalian health: 17α-estradiol, acarbose, green tea extract, nordihydroguaiaretic acid, and rapamycin. We found that green tea extract and nordihydroguaiaretic acid extend *Caenorhabditis* lifespan in a species-specific manner. Additionally, these two antioxidants conferred assay-specific effects in some studies—for example, decreasing survival for certain genetic backgrounds in manual survival assays in contrast with extended lifespan as assayed using automated *C. elegans* Lifespan Machines. We also observed that GTE and NDGA impact on older adult mobility capacity is dependent on genetic background, and that GTE reduces oxidative stress resistance in some *Caenorhabditis* strains. Overall, our analysis of the five compounds supports the general idea that genetic background and assay type can influence lifespan and health effects of compounds, and underscores that lifespan and health can be uncoupled by chemical interventions.

**Supplementary Information:**

The online version contains supplementary material available at 10.1007/s11357-023-00978-0.

## Introduction

Elaboration of clinical strategies that delay health declines associated with aging is anticipated to markedly improve life quality for the elderly and their families. One research area focused on such a goal seeks to identify compound interventions that can prolong life and/or promote healthy aging. Toward this end, model organisms are invaluable in initial compound screening as these models offer low expense, ease of culture, short lifespans, and often simple health assays. One of the most widely studied aging models, *Caenorhabditis elegans*, has proven a useful system for identifying and characterizing compounds that robustly extend lifespan and healthspan [1–3].

The *Caenorhabditis* Intervention Testing Program (CITP), an NIH-funded research consortium consisting of investigators at three independent sites (Rutgers University, the University of Oregon, and the Buck Institute for Research on Aging), is tasked to identify pharmacological interventions with the potential to extend *Caenorhabditis* lifespan and healthspan in a robust manner. The founding principle for the CITP is that compounds with positive effects across a genetically diverse population engage conserved biochemical pathways that promote healthy aging. The CITP is distinctive in testing compounds across a genetically diverse panel of *Caenorhabditis* strains and species to identify interventions that promote lifespan extension independent of genetic background [4]. Another distinctive feature of the CITP effort is that studies are replicated as closely as possible at the three geographically distinct sites. To date, the CITP has reported on the lifespan and healthspan effects of more than 27 compounds in more than 250,000 individuals across nearly 280 trials [5–10].

The initial CITP studies focused on longevity as the sole endpoint for anti-aging intervention evaluation [5–8]. For the current suite of test compounds, we expanded and modified our workflow [5] to evaluate the potential of compounds to extend lifespan and/or healthspan across *Caenorhabditis* species and strains (Supplemental Fig. 1).

Here, we report results from five compounds in the CITP testing pipeline: 17α-estradiol, acarbose, green tea extract (GTE), nordihydroguaiaretic acid (NDGA), and rapamycin. 17α-estradiol, a weak endogenous steroidal estrogen, has been reported to alleviate age-related metabolic dysfunction and inflammation in male mice [11], to protect against neuro-degeneration in cell and animal models of Parkinson’s disease [12], and to extend lifespan in genetically heterogenous male mice [13, 14]. Acarbose, an anti-diabetic drug that inhibits alpha-glucosidase, has been shown to prevent age-related glucose intolerance [15] and to limit postprandial hyperglycemia in mice [16, 17]. Likewise, acarbose has been shown to extend median lifespan in genetically heterogenous mice [13, 14]. GTE is rich in antioxidant polyphenols, and has been reported to reduce the risk of coronary heart disease and certain forms of cancer [18], as well as to provide neuroprotection against diseases such as Alzheimer’s [19]. GTE increases lifespan in both flies [20, 21] and mice [22], and its primary constituent flavonoid, epigallocatechin gallate (EGCG), has been shown to extend mean lifespan in *C. elegans* [23], although that result may be context dependent [24, 25]. NDGA, a lignin found in the creosote bush, possesses both antioxidant and anti-inflammatory properties [26, 27], and has been shown to increase lifespan in mice [13, 28]. Finally, rapamycin, an mTOR kinase inhibitor, has been shown to increase lifespan in a variety of model organisms, including mice and flies [29–31].

Our studies of 17α-estradiol, acarbose, GTE, NDGA, and rapamycin underscore the complexities of assessing biological outcomes of candidate lifespan extending treatments. Using our standardized protocols, we find that the antioxidants GTE and NDGA extend *Caenorhabditis* lifespan in a species-specific manner. GTE and NDGA tests also revealed some assay-specific outcomes—in certain genetic backgrounds we found decreased survival in manual longevity assays, whereas we measured extended lifespan when we determined outcomes using the automated *C. elegans* Lifespan Machines (ALM). GTE and NDGA affected swimming ability in a strain-specific manner, and GTE lowered oxidative stress resistance in some *Caenorhabditis* strains. Lifespan and healthspan could thus be uncoupled as evaluated by our approach, with outcomes influenced by genetic background. Overall, our findings on this test set of interventions underscore how impactful genetic background, selected health assay, and protocol details are in the assessment of intervention effects. Interventions that meet the high bar of efficacy across a broad range of genetic backgrounds and across multiple experimental approaches may prove the exception but would establish definitive priority for testing in mammalian models.

## Materials and methods

### Strains

All natural isolates used were obtained from the *Caenorhabditis* Genetics Center (CGC) at the University of Minnesota: *C. elegans* N2, MY16, and JU775; *C. briggsae* AF16, ED3092, and HK104; *C. tropicalis* JU1630, JU1383, and QG834. Worms were maintained at 20 °C on 60 mm NGM plates seeded with *Escherichia coli* OP50-1.

### Interventions

Compound intervention treatments were performed as previously described [5]. The compounds used include green tea extract (LKT Laboratories, Inc. G6817, lot #2595901), nordihydroguaiaretic acid (Sigma-Aldrich 74540, lot #BCBQ4489V), α-estradiol (Sigma-Aldrich E8750, lot #016M4175V), rapamycin (LC laboratories R-5000, lot #ASW-135), and acarbose (Sigma-Aldrich A8980, lot #MKBS1059V0). Compounds were obtained as solids and dissolved in either water or DMSO (dimethyl sulfoxide) to obtain stock solutions, with either water or DMSO used to treat control agar plates. DMSO stock solutions (both compound and control) were then further diluted with water to create working solutions to allow for even distribution across the agar plate while maintaining a final concentration of 0.25% DMSO. Agar plates were treated with compound stock solutions such that the final volume was assumed equal to the volume of the agar.

### Manual lifespan assay

Per the previously published CITP standard operating procedure [5], synchronized populations were generated via timed egg lays on 60 mm NGM plates. At day one of adulthood, 50 worms were transferred to 35 mm NGM plates containing 51 µM FUdR and compound intervention (or the solvent control). Worms were then transferred to fresh plates and scored as alive or dead on day two and five of adulthood for *C. elegans* and *C. briggsae*, or day two and four of adulthood for *C. tropicalis*. Thereafter, worms were transferred once weekly and scored every Monday, Wednesday, and Friday until dead. Death was defined as a lack of response when stimulated with a platinum wire.

### Automated lifespan assay

ALM assays were performed as previously described [6, 32, 33], based on modification of the protocols published for the Lifespan Machine [34]. Briefly, worms were age synchronized and transferred to intervention plates as described above. One week post egg lay, 50 animals were transferred to 50 mm tight-lidded, intervention treated, modified NGM plates containing 51 µM FUdR and 100 µM nystatin and loaded onto the ALMs. Scanner data was collected and analyzed using the Lifespan Machine software (https://github.com/nstroustrup/lifespan; [34] and strain-specific posture files [6]).

### Swimming ability assay

Swimming ability was measured per the standard CITP protocol [35, 36], using the *C. elegans* Swim Test system (CeleST) [37, 38]. Worms were age-synchronized and exposed to compound intervention during adulthood as described above, until swimming measurements were collected at ages 6 and 12 of adulthood (*C. elegans* and *C. tropicalis*), or ages 8 and 16 of adulthood (*C. briggsae*). Videos were processed using the CeleST software (https://github.com/DCS-LCSR/CeleST).

### Thermotolerance assay

The ability for animals to withstand heat stress was measured as previously published [35], utilizing a modification of the ALM protocol. Worms were synchronized and aged as adults on compound intervention plates, as stated above, until the desired testing age (adult day 6 and 12 for *C. elegans* and *C. tropicalis*, days 8 and 16 for *C. briggsae*). At this time, animals were placed onto 50 mm plates with modified NGM containing 100 µM nystatin, without FUdR or compound intervention, at a density of 70 worms per plate. Plates without lids were then transferred to Automated Lifespan Machines in an incubator set to 32 °C and 50% humidity. Scanner data were collected using an increased scan speed and reduced resolution to provide proper temporal resolution, needed as a result of the shortened survival under these conditions. Images analyzed using the Lifespan Machine software (https://github.com/nstroustrup/lifespan) [34] and strain-specific posture files [6] and deaths were validated by hand.

### Oxidative stress resistance assay

Resistance to oxidative stress was measured per the standard CITP procedure [35]. Worms were prepared and aged as adults on interventions as mentioned above, with the same ages tested as in the swim test and thermotolerance assays. At the desired age, animals were transferred at a density of 70 worms per plate to 50 mm tight-lidded plates with modified NGM containing 40 mM paraquat (or methyl viologen dichloride, from Sigma-Aldrich), 51 µM FUdR, 100 µM nystatin. Plates were then transferred to ALMs at 20 °C and scanner data collection, processing, and analyzing were done with the same methodology mentioned for the thermotolerance assay.

## Results

### The CITP pipeline for the evaluation of compounds on lifespan and healthspan

**Fig. 1:**
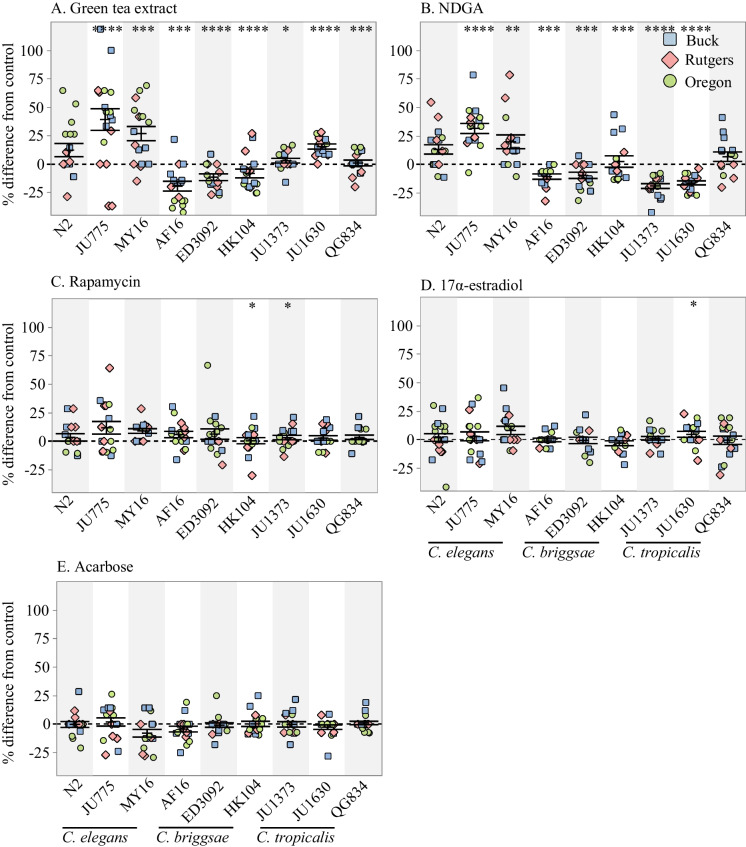
The effect of adult exposure to **a** 500 μg/mL green tea extract, **b** 100 μM NDGA, **c** 50 μM rapamycin, **d** 30 μM 17α-estradiol, and **e** 500 μM acarbose on the median lifespan of nine strains across three *Caenorhabditis* species. Three strains were tested from each species: *C. elegans* N2, MY16, and JU775 (black text), *C. briggsae* AF16, ED3092, and HK104 (dark gray text), *C. tropicalis* JU1373, JU1630, and QG834 (light gray text). Each point represents the change in median lifespan from an individual trial plate (compound treated) relative to the specific control (vehicle only) conducted. The bars represent the mean + / − the standard error of the mean. Replicates were completed at the three CITP testing sites (blue square–Buck Institute, green circle–Oregon, and salmon diamond–Rutgers). Asterisks represent *p*-values from the CPH model such that ****p < 0.0001, ***p < 0.001, **p < 0.01, and *p < 0.05

**Fig. 2:**
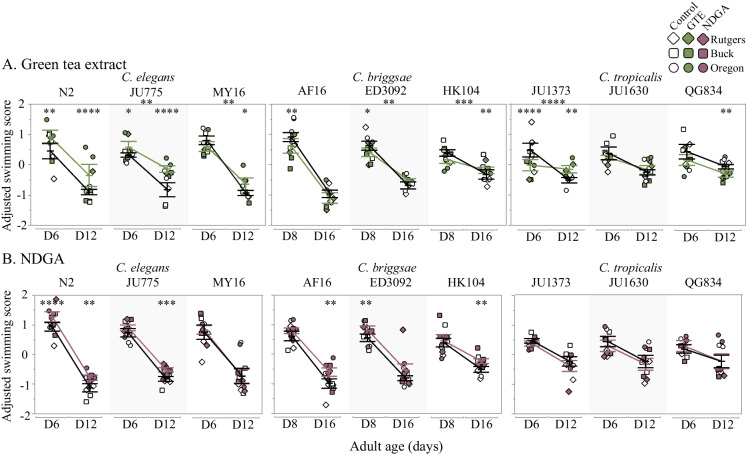
The mean adjusted swimming score across *Caenorhabditis* species after treatment with **a** GTE, or **b** NDGA is shown for young and old ages (adult days 6 and 12 for *C. elegans* and *C. tropicalis*, adult days 8 and 16 for *C. briggsae*). Strains tested include the following: three *C. elegans* strains (N2, JU775, MY16), three *C. briggsae* strains (AF16, ED3092, HK104), and three *C. tropicalis* strains (JU1373, JU1630, QG834). Each data point represents the mean from one trial, the bars represent the mean + / − the standard error of the mean, and the colors correspond to the treatment conditions starting at day 1 of adulthood (white–vehicle control, green–500 ug/mL GTE, mauve–100 uM NDGA). Replicates were completed at the three CITP testing sites (square- Buck Institute, diamond- Rutgers, circle- Oregon). Asterisks inside the plots represent *p*-values from the linear mixed model, and asterisks outside the plot represent *p*-values from the type III ANOVA indicating a significant compound by age interaction, such that ****p < 0.0001, ***p < 0.001, **p < 0.01, and *p < 0.05

**Fig. 3:**
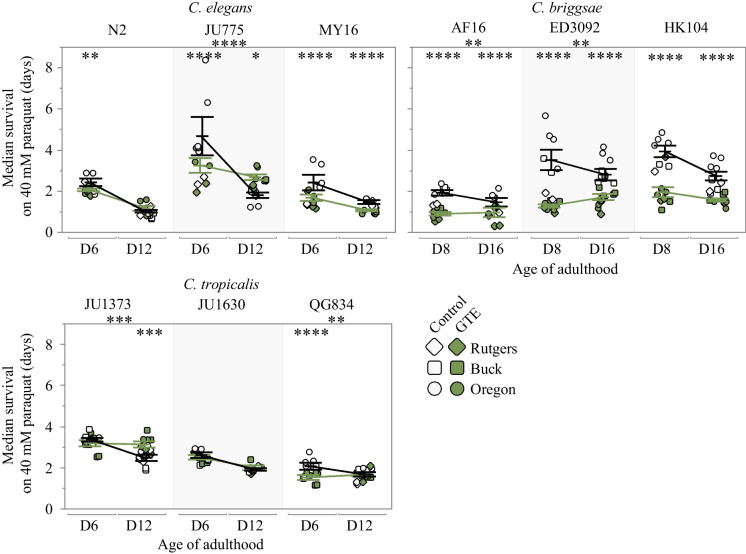
Survival of nine *Caenorhabditis* strains on 40 mM paraquat after adult exposure to green tea extract (green symbols), or vehicle control (open symbols). Oxidative stress resistance was measured at day 6 and 12 of adulthood (*C. elegans* and *C. tropicalis*), or day 8 and 16 of adulthood (*C. briggsae*). Three strains were tested from each species: *C. elegans* N2, JU775 and MY16, *C. briggsae* AF16, ED3092 and HK104, and *C. tropicalis* JU1373, JU1630, and QG834. Dots represent the median survival of one plate replicate (gray–vehicle only control, green–500 μg/mL GTE). Replicates were completed at three CITP testing sites (circle–Oregon, square–Buck, diamond–Rutgers). Asterisks inside the plots represent *p*-values from the CPH model, and asterisks outside the plot represent *p*-values from the type III ANOVA indicating a significant compound by age interaction, such that ****p < 0.0001, ***p < 0.001, **p < 0.01, and *p < 0.05

We created a workflow plan to evaluate the ability of a chemical intervention to extend lifespan and/or healthspan across *Caenorhabditis* species and strains (Supplemental Fig. 1). The CITP pipeline involves preliminary testing in a single lab to identify a potentially effective dosage range (phase one study; Supplemental Fig. 2) and to target a bioactive dose across strains (phase two; Supplemental Fig. 3) before we undertake survival assays in all three CITP labs (phase three; Fig. 1). In phase four, we evaluate the effect of bioactive compounds that passed the preliminary tests for robust impact on longevity (phases 1–3) on select health measures (Figs. 2 and 3). We publish our findings on compounds without positive lifespan that exit the pipeline [7–9] (Supplemental Fig. 1).

### The antioxidants green tea extract and NDGA extend *Caenorhabditis* lifespan in a species-specific manner

Using the streamlined pipeline we describe above, we evaluated a test set of compounds for lifespan effects. Among this test set, we found that rapamycin, acarbose, and 17α-estradiol were broadly ineffectual. Rapamycin only altered lifespan for two (*C. briggsae* HK104 and *C. tropicalis* JU1373) of the nine tested strains, while 17α-estradiol only had an effect on JU1630 (Fig. 1). We also found that acarbose did not statistically change the lifespan of any of the tested strains.

The two antioxidants we tested, GTE and NDGA, exerted the largest effect on lifespan in manual survival assays (Fig. 1). GTE extended lifespan in two species, *C. elegans* (two of three strains) and *C. tropicalis* (all three strains) (Fig. 1a). The effect on lifespan was more pronounced in *C. elegans*, with mean lifespan extended > 15% in both MY16 and JU775. In the laboratory *C. elegans* strain N2, we observed lifespan extension via GTE in only one lab, and the pooled results across labs was insignificant. We also observed lifespan extension with GTE in *C. tropicalis*, with all three strains tested showing small but significant increases in mean lifespan; the largest effect was in JU1630 (~ 11% increase in mean lifespan). In contrast, we found that all *C. briggsae* strains exhibited a significant decrease in survival when exposed to GTE, with mean lifespan decreasing by 10–19%. Overall, we observe a strong species-specific effect on longevity with GTE exposure.

We found that NDGA also impacted *Caenorhabditis* longevity in a species-specific manner (Fig. 1b). In *C. elegans*, much like with GTE, we measured an 8–16% increase in the mean lifespan of strains MY16 and JU775, while N2 had a similar, albeit insignificant, lifespan increase. The effect of NDGA on lifespan was more variable than we recorded for GTE as we observed a significant decrease in survival in five of six *C. briggsae* and *C. tropicalis* strains.

### Antioxidants that decrease survival in certain genetic backgrounds extend lifespan in automated *C. elegans* Lifespan Machine assays

To increase throughput, we have implemented ALMs [34] (Supplemental Fig. 1, Phase Two) at all three CITP sites. The ALM is a lifespan analysis platform built on flatbed scanner technology that enables life-long imaging of animals at 1-h intervals, increasing both throughput and temporal resolution for data sampling. Our previous testing of compounds NP1, resveratrol, propyl gallate, thioflavin t, and α-ketoglutarate revealed that most ALM trials recapitulated outcomes from manual plate-based assays, although we did identify light sensitivity of some compounds as a factor that could change outcome [6]. During preliminary experiments on ALMs, we found that both GTE and NDGA increased lifespan in strains that also reported decreased lifespans in manual assays. More specifically, for GTE assays on the ALM, all *C. briggsae* strains exhibited increased survival compared to the control. For NDGA treatment, four of the six *C. briggsae* and *C. tropicalis* strains exhibited positive lifespan results on the ALM. Difference in outcome might be the consequence of increased light exposure on the ALMs [32], as light is known to induce photooxidative stress [39]. ALM protocols also involve fewer potentially damaging animal transfers, and feature no exposure to freshly treated compound plates in mid- to late-life, in contrast to manual assays [33, 40]. Our highly replicated data, however, emphasize that the methodological approach to lifespan determination is a factor in experimental outcome.

### GTE and NDGA impact on swimming ability is strain-specific

With the goal of maintaining health over the lifetime, compounds that slow the age-related decline in swimming ability are of particular interest to the CITP. We previously showed that swimming ability as a measure of locomotion with compound treatment in *Caenorhabditis* does not always correlate with lifespan, and thus swim locomotion is an assay with potential to identify compounds that may improve healthspan independent of lifespan [35]. Because both GTE and NDGA induced strong species-specific effects on lifespan, we addressed whether these compounds could improve locomotion.

We found that with GTE treatment, all *C. elegans* strains showed an improvement in swimming ability (Fig. 2a). The improved locomotion effect was age-dependent in MY16 and JU775 (8–9% increase in mean swim score at old age as compared to the control, respectively), while N2 showed an overall robust improvement in locomotion (25% increase at young age, 146% increase at old age). The *C. tropicalis* strains (in which GTE improved lifespan), exhibited strain-specific responses in swimming ability: strain JU1630 showed no difference in locomotion with GTE treatment, while strain QG834 exhibited a small but significant decrease in swimming ability (2–4% decrease with age). JU1373 exhibited a strong age-related response in which GTE robustly decreased swimming ability at young age (14% decrease) but improved swimming ability during old age (16% increase). The effect of GTE on swimming ability in *C. briggsae* was minimal but strain-specific as we recorded decreases in swimming ability at young age in strains AF16 (23% decrease) and ED3092 (13% decrease), but a small increase at old age in HK104 (6% increase).

We also tested the effect of NDGA on locomotion in our panel of *Caenorhabditis* strains. With NDGA treatment, swimming ability improved in five of six *C. elegans* and *C. briggsae* strains, but none of the *C. tropicalis* strains (Fig. 2b). The effect in *C. elegans* was observed in two strains, with N2 gaining a general increase (11–13% increase in mean swimming with age) and with JU775 exhibiting a reduction in the age-related decline of swimming ability (6% increase at old age). Interestingly, all three *C. briggsae* strains showed an age-dependent improvement of locomotion at either young age (ED3092, 6% increase), or old age (HK104 and ED3092, 19–21% increase, respectively), irrespective of their reduced lifespan with NDGA treatment. *C. tropicalis*, which showed either no effect or a negative lifespan effect in response to NDGA, had no difference in swimming ability with NDGA treatment.

Overall, our tests underscore the following: (1) longevity and locomotory health are not well correlated; (2) interventions can elicit marked differences in locomotory healthspan, even in the absence of longevity changes.

### GTE reduces oxidative stress resistance in some *Caenorhabditis* strains

We next assayed oxidative stress resistance with GTE treatment across our panel of strains because GTE exerted the most widespread effect on longevity and locomotion of the two antioxidants tested. In seven of the nine strains, GTE treatment reduced the animal’s ability to resist oxidative stress for at least one age tested (Fig. 3). This effect was most prominent in the *C. briggsae* strains, which exhibited significant decreases in survival at all ages tested (ranging from a 17% decrease in mean survival in old AF16 to a 65% decrease in young ED3092). Previous exposure to a mild stressor can result in a more robust response to future stressors, a process known as hormesis [41]. Continuous exposure to an antioxidant may subsequently reduce reactive oxygen species that promote an organism’s ability to mount an enhanced oxidative stress response when removed from the compound. While GTE does have antioxidant properties [42], the inability to resist oxidative stress may reflect an anti-hormetic effect. The only exceptions to the failure of GTE to increase oxidative stress resistance were in *C. tropicalis* JU1373, which exhibited an age-dependent increase in oxidative stress survival (22% increase at old age), and JU1630, with no effect at either age. *C. elegans* JU775 also had an increase in oxidative stress survival as compared to the control during old age (35% increase), though this increase was paired with a decrease in oxidative stress survival at young age (31% decrease).

We have found ALM-based thermotolerance assays to be challenging to reproduce across labs [35]. Still, as we continue to assess how broadly across compounds health measures are impacted, we tested thermotolerance consequent GTE treatment. As we previously observed, there was a high amount of variability within our dataset that made it difficult to identify significant effects on thermotolerance, but our results suggest that GTE may protect against thermosensitivity as most of the strains exhibited lifespan extension with GTE treatment (Supplemental Fig. 4).

## Discussion

### Examples of interventions that exert varied effects across species

As a group dedicated to identifying highly reproducible pharmacological interventions that extend lifespan, promote fundamental functionality such as locomotory ability, or both, with efficacy that applies over a genetically diverse Caenorhabditis test set, the CITP has first focused on testing compounds published to be effective in extending lifespan in nematodes or other model systems [5–8]. The five compounds we studied here, 17α-estradiol, acarbose, green tea extract, nordihydroguaiaretic acid, and rapamycin, are representative of that interest class of interventions. We evaluated the five compounds for longevity modulation across a genetically diverse test set and pursued two of the more potent promoters of longevity, GTE and NDGA, for impact on locomotory health and stress resistance. We observed that the antioxidants GTE and NDGA modestly extend *Caenorhabditis* lifespan in a species-specific manner (Fig. 1). Additionally, we found that two different survival assay protocols we employed (manual and using Automated Lifespan Machines) could report different lifespan outcomes of these antioxidants, with observed decreased survival for certain genetic backgrounds in manual survival assays contrasting with extended lifespan as determined on the ALMs (Fig. 1 and Supplemental Figs. 2, Fig. 3). GTE and NDGA confer strain-specific impact on swimming ability (Fig. 2), although GTE reduces oxidative stress resistance in some *Caenorhabditis* strains (Fig. 3), suggesting that GTE targets and the underlying cause(s) of diminished health for these assessments may reflect different processes. While lifespan and health can certainly be uncoupled, and both are plausible targets for intervention, this study, combined with previous observations, underscores the complex challenge to finding universal lifespan and healthspan extending interventions.

### Implications for identification of conserved pharmacological interventions

Aging is a complex trait with a broad range of associated measurable outcomes. The inter-individual variability in outcome necessitates studies of significant size and replicate number to reliably identify anti-aging interventions. Because of the necessary study sizes, and the need to follow individuals over the lifetime, the initial characterization of compound effects on aging is frequently performed in short-lived animal models. The oft-unspoken assumption is that the near universality of phenotypic aging in multi-cellular animals reflects shared underlying causes of aging, and insights gained in model systems will be generalizable across taxa. At the same time, genetically identical individuals in the same environment can exhibit strikingly different aging trajectories [5, 43, 44]. Whether that variability in outcome reflects differences in aging symptoms due to unidentified environmental differences or stochastic biological events or, importantly, if those differences reflect different causes of aging experienced by individuals is not known.

The CITP attempts to account for issues of inter-individual differences and stochastic events by testing compounds at a large experimental scale using standardized protocols [33, 40], replicating experiments at three independent sites, and statistically partitioning the variance to determine the sources of variability (Supplemental Table 1 for this study supports that lab specific differences account for very little of experimental variation for GTE lifespan). The CITP tests compound interventions across a genetically diverse panel of *Caenorhabditis* strains and species to address the potential to identify interventions that are effective independent of genetic background. Presumably such broadly acting compounds would engage widely conserved pro-longevity mechanism. The large number of animals studied, and the numbers of independent replicates in CITP assays, increase confidence in reported outcomes.

In reporting outcomes, CITP always emphasizes that our conclusions, although rigorously reproduced, apply under the specific experimental conditions used for our investigation. For any reported CITP outcome, it remains possible that assay under alternative conditions will lead to distinct conclusions. Factors that lead to differences among studies conducted in the field include compound concentration and source, precise media composition, use or not of FUdR to induce sterility, and period of exposure to the intervention. As noted above, in this study, we also document an example in which method of analysis—manual vs. automated longevity assays can produce markedly different outcomes. Furthermore, when testing against a genetically diverse population as is modeled by CITP, the challenge of finding the optimal intervention introduction conditions is amplified by a need for chemical uptake by all strains and metabolism that will engage in similar ways. These challenges, and the expectation that many false negative outcomes will result, are hypothesized to be overcome by the identification of a rare broadly efficacious compound. We did not identify such a compound in the small set of 5 compounds tested in this study.

### Regarding the challenge of translation and implications for identifying compounds that slow aging using *C. elegans*

Considerable interest in the application of TOR inhibitor Rapamycin for pro-longevity outcomes, anchored in both invertebrate and mammalian models, exists in the aging field [45–47] and strong genetic data support the importance of mTOR in longevity pathways under stress. That rapamycin was largely ineffective under CITP assay conditions may reflect one of the many conceptual impediments to direct translation success noted above, including intervention bioavailability and uptake, metabolism, and particular endpoints selected for assessment. Because of these considerations, we are careful to point out that absence of effect does not rule out that a compound might work in mammals.

Rather, it is the rare compound that passes out of the rigorous CITP testing that we seek to highlight for translational consideration. Compounds that are effective in promoting longevity in *C. elegans* and mammals have been reported. Examples of pharmacological interventions identified by *C. elegans* screening for longevity include anti-hypertension drug Captropil [48] which extended longevity in female mice [49]. Anti-diabetic drug Metformin can promote longevity in *C. elegans* and locomotory health in both *C. elegans* and *C. briggsae* [10] and promotes health and longevity in some mammalian studies [50, 51]. In this study, NDGA, which can extend longevity in male mice [28], extends lifespan across elegans variants.

As CITP generates additional data, cataloging successes in parallel mammalian outcomes will be important for honing screens to that best prioritize compounds for likelihood of benefit in mammals. Possibly, however, a strict adherence to a longevity endpoint is too narrow a net to cast. Along those lines, we note that collective data are converging to suggest the underappreciated value of the extent to which identified longevity compounds for *C. elegans* promote mammalian health and healthy aging. For example, CITP identified Thioflavin T as highly potent in *C. elegans* for longevity and mobility [5]. Testing of a more mouse-friendly variant of Thioflavin T, 2-hydroxyphenyl-benzoxazole, revealed a remarkable improvement in old age bone maintenance in mice [52] and protection against ALS pathology [53]. The geroscience concept (since aging predisposes to disease, interventions that target aging will also be effective against disease [54]) predicts such an outcome—interventions that robustly extend lifespan in *C. elegans* (genetic diversity similar to that among humans) or in *Caenorhabditis* species (genetic diversity similar to the mouse to human difference) may often confer the capacity to protect against disease and specific conditions of aging in all animals.

### Health measures and longevity

The important question of how longevity and health intersect and are controlled by “aging pathways”, and the connection between them, is a matter of controversy [44]. The question of pharmacological impact on health in the context of genetic diversity has not been well investigated in models, despite the importance for considerations of translation in human populations.

Here we monitored *Caenorhabditis* locomotory features at multiple adult ages to conclude that there is not a strong correlation between longevity and locomotion for the five test compounds of focus. Moreover, assays of oxidative stress resistance and heat resistance did not establish a relationship between longevity and these health indicators across genetic backgrounds. Perhaps an important take home message for health metrics is that often-used metrics of stress resistance are anchored in acute, and likely non-physiological, challenges (i.e., 40 mM paraquat) that might be replaced by assays that more closely approximate native challenges of aging. For example, use of stress reporter gene expression patterns might prove to correlate better with animal health and longevity, providing a more precise assessment tool for intervention potential.

Against a small but expanding test set, we observe that compound genetic background and assay type can give rise to differences in lifespan evaluation and health assessment [5, 6]. The disconnect between lifespan and health measurements, and the genetic background dependency of those effects suggests additional complications that need to be addressed in screening experimental design. Stepping back to offer some perspective, it is likely unrealistic to expect many blockbuster aging interventions using broad based outcome evaluation. Nonetheless, the pursuit of the particular compounds that can positively move outcomes across genetic backgrounds and via multiple measures of heath remains a plausible strategy that spotlights priority interventions for mammalian testing.

## Conclusion

Our study of five interventions (NDGA, GTE, 17α-estradiol, acarbose, and rapamycin) underscore the complexities of assessing longevity and health outcomes of candidate aging interventions. Using our standardized protocols, we find that the antioxidants GTE and NDGA extend Caenorhabditis lifespan in a species-specific manner. GTE and NDGA tests also revealed some survival assay-specific outcomes—in certain genetic backgrounds we found decreased survival in manual longevity assays, whereas we measured extended lifespan when we determined outcomes using the automated *C. elegans* Lifespan Machines. GTE and NDGA affected swimming ability in a strain-specific manner, and GTE lowered oxidative stress resistance in some *Caenorhabditis* strains. Lifespan and healthspan generally appear uncoupled in our dataset. Overall, our findings on this test set of interventions underscore how impactful genetic background, selected health assay, and protocol details are in the assessment of intervention effects. Interventions that meet the high bar of efficacy across a broad range of genetic backgrounds and across multiple experimental approaches may prove the exception, but such capacity would establish definitive priority for testing in mammalian models.

### Supplementary Information

Below is the link to the electronic supplementary material.

Supplementary Fig. 1 (JPG 168 KB)

Supplementary Fig. 2 (JPG 292 KB)

Supplementary Fig. 3 (JPG 335 KB)

Supplementary Fig. 4 (JPG 144 KB)

Supplementary Table 1 (JPG 103 KB)

## Data Availability

All data, analysis scripts, and detailed SOPs are available as an online supplement [55].
