# Supplementary figures and images for: Antioxidants green tea extract and nordihydroguaiaretic acid confer species and strain-specific lifespan and health effects in Caenorhabditis nematodes
Source: GeroScience. 2023 Nov 4;46(2):2239–51. doi: 10.1007/s11357-023-00978-0 (PMC10828308; doi:10.1007/s11357-023-00978-0)

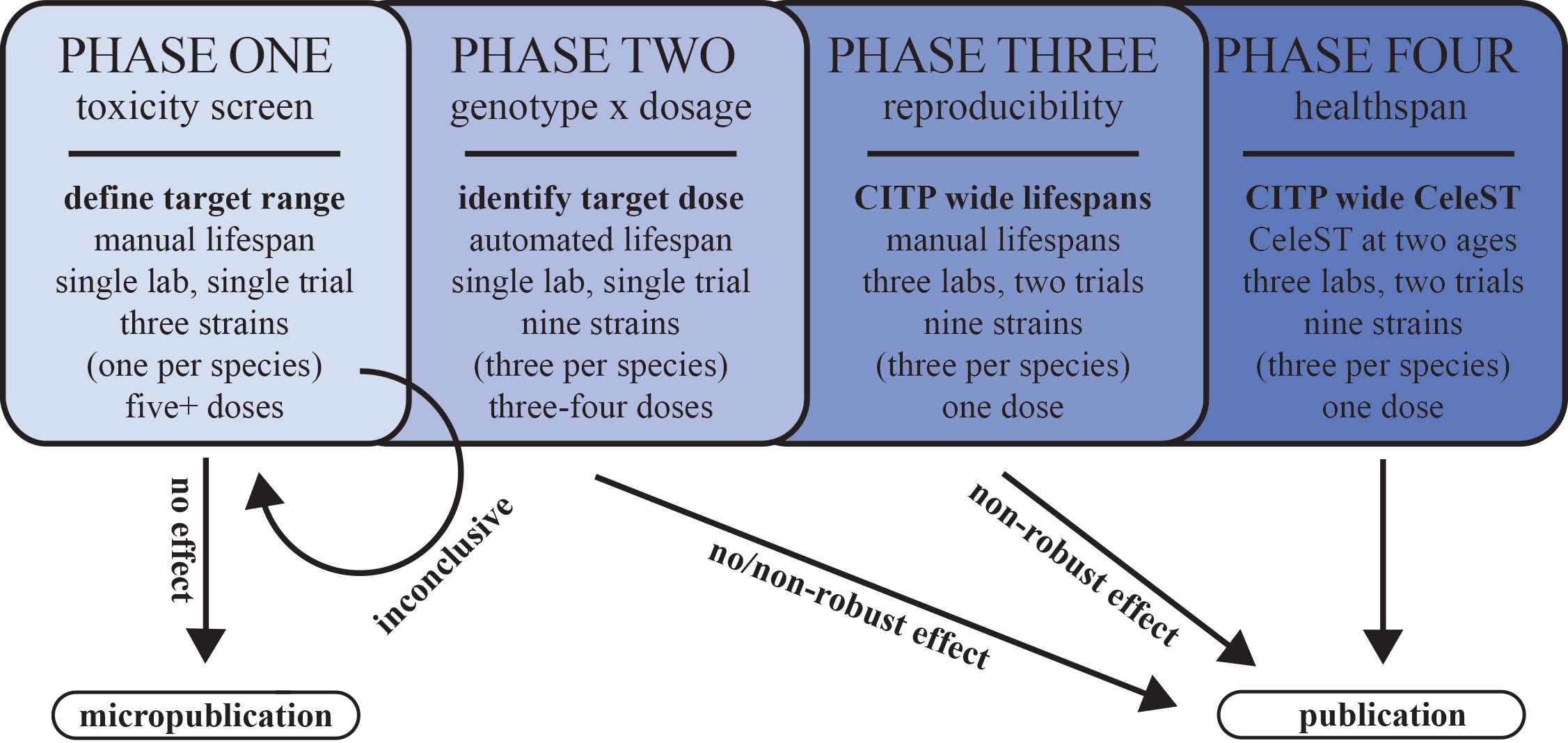

Supplement: Supplementary file 1 — Supplementary Fig. 1 (JPG 168 KB) [file 11357_2023_978_MOESM1_ESM.jpg]

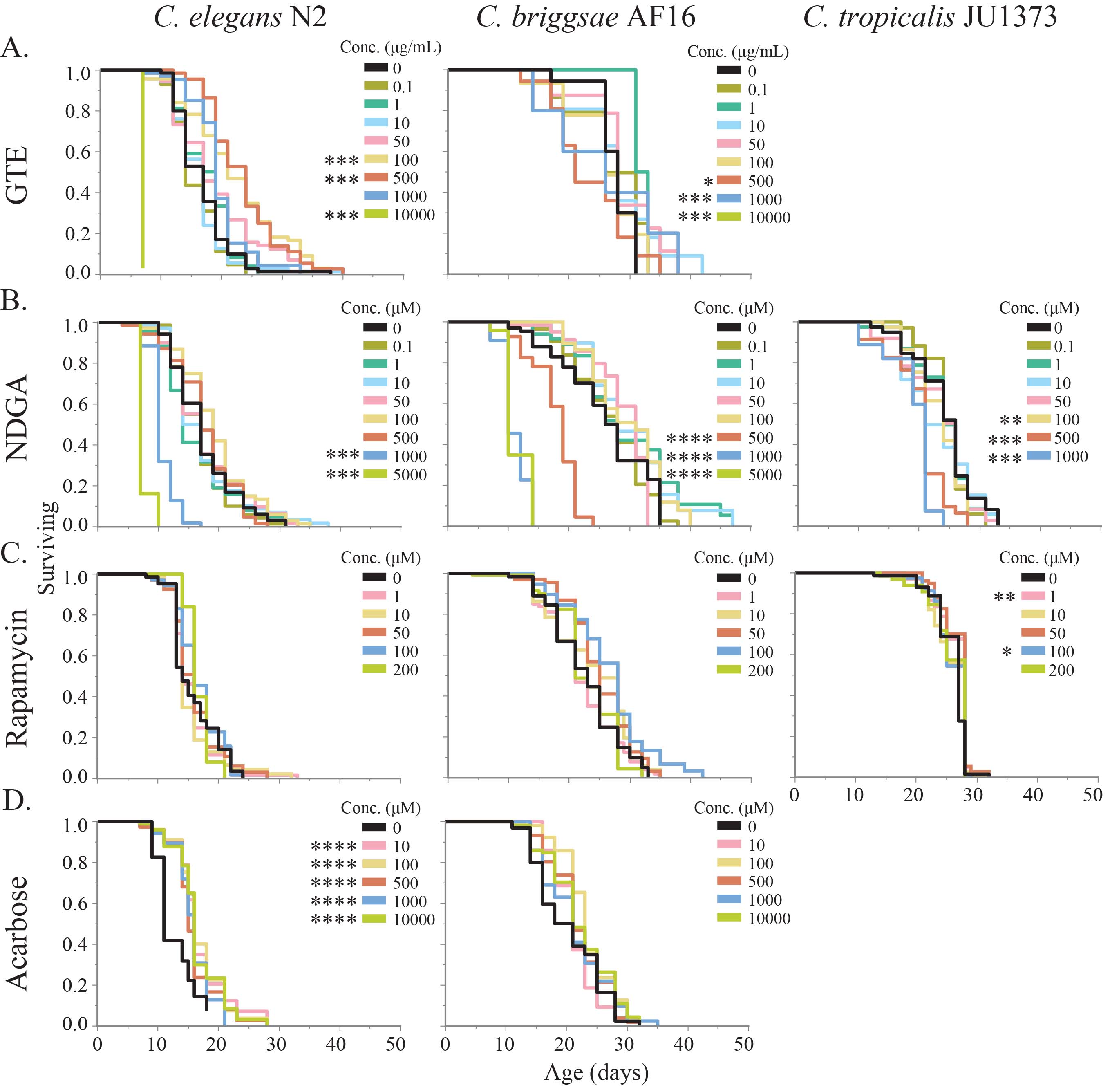

Supplement: Supplementary file 2 — Supplementary Fig. 2 (JPG 292 KB) [file 11357_2023_978_MOESM2_ESM.jpg]

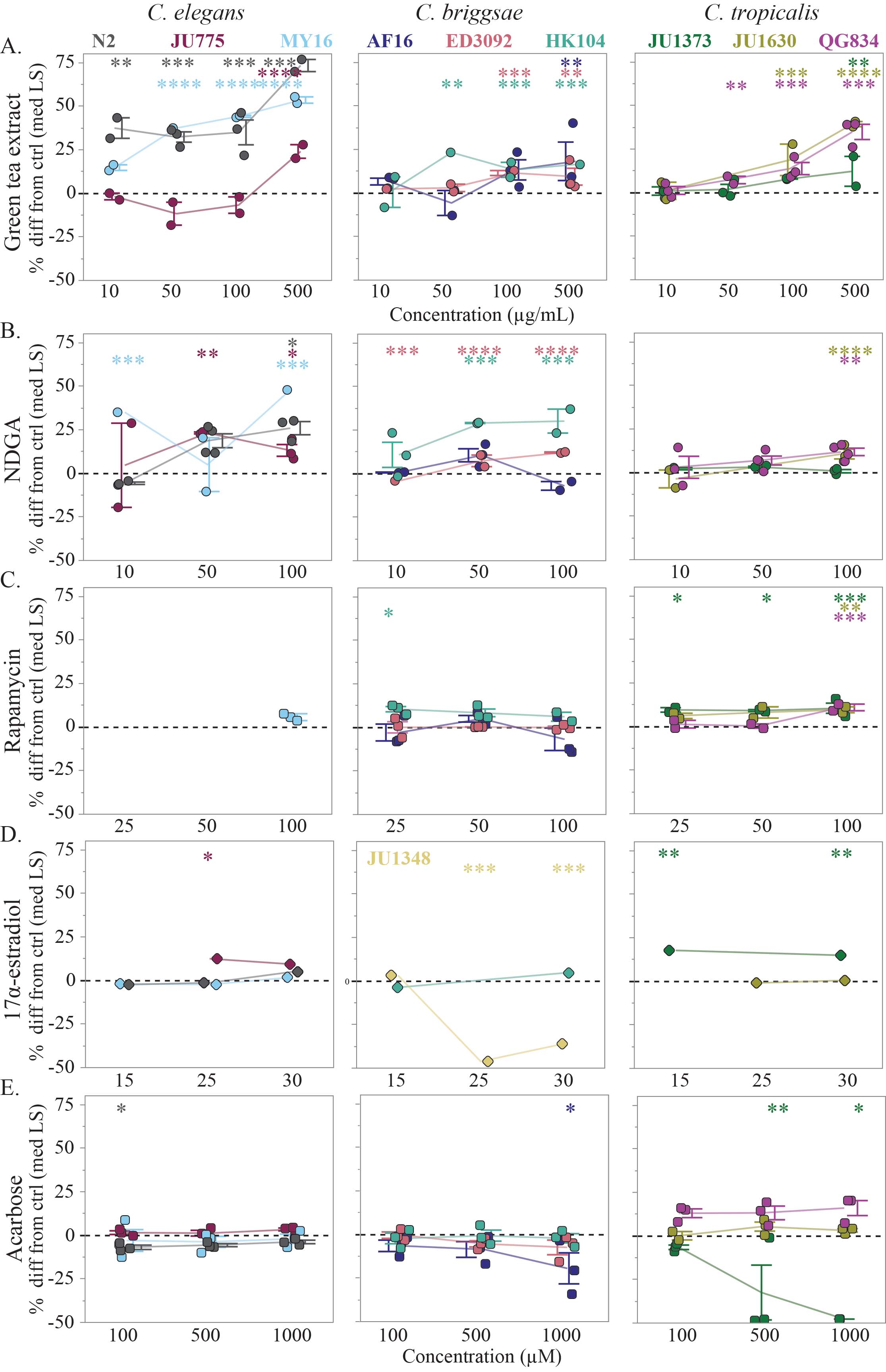

Supplement: Supplementary file 3 — Supplementary Fig. 3 (JPG 335 KB) [file 11357_2023_978_MOESM3_ESM.jpg]

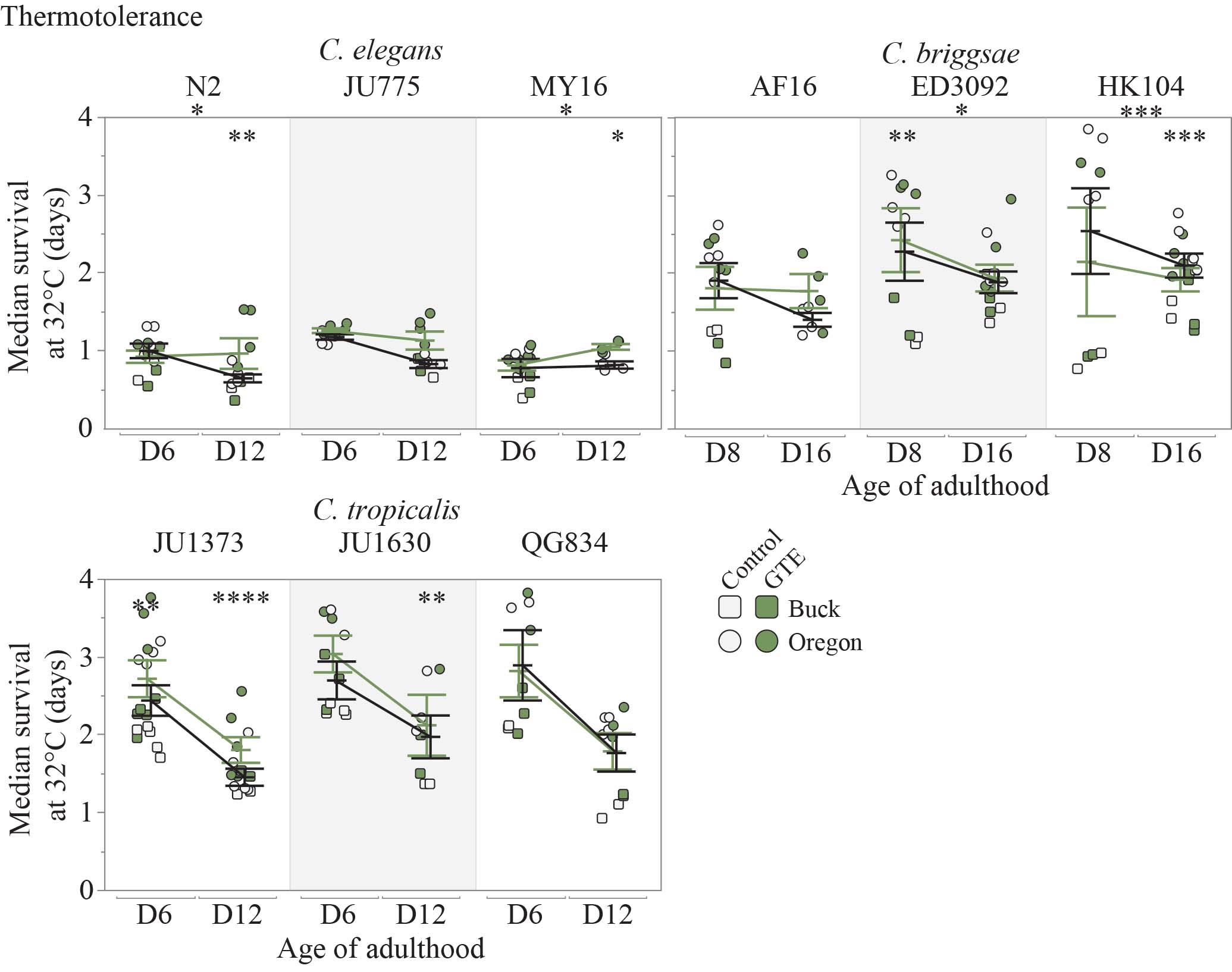

Supplement: Supplementary file 4 — Supplementary Fig. 4 (JPG 144 KB) [file 11357_2023_978_MOESM4_ESM.jpg]

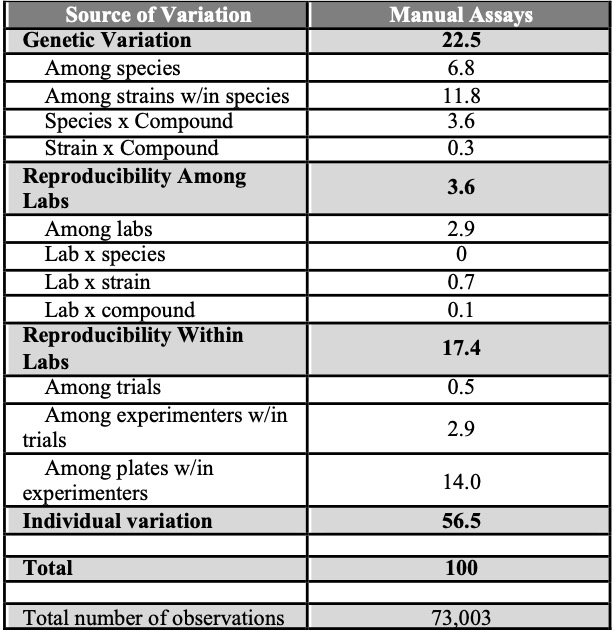

Supplement: Supplementary file 5 — Supplementary Table 1 (JPG 103 KB) [file 11357_2023_978_MOESM5_ESM.jpg]
